# Progesterone receptor activation downregulates GATA3 by transcriptional repression and increased protein turnover promoting breast tumor growth

**DOI:** 10.1186/s13058-014-0491-x

**Published:** 2014-12-06

**Authors:** Franco Izzo, Florencia Mercogliano, Leandro Venturutti, Mercedes Tkach, Gloria Inurrigarro, Roxana Schillaci, Leandro Cerchietti, Patricia V Elizalde, Cecilia J Proietti

**Affiliations:** 1 Instituto de Biología y Medicina Experimental (IBYME), CONICET, Vuelta de Obligado 2490, Buenos Aires, 1428 ADN Argentina; 2 Sanatorio Mater Dei, Buenos Aires, Argentina; 3 Weill Cornell Medical College, New York, 10021 NY USA

## Abstract

**Introduction:**

The transcription factor GATA3 is involved in mammary gland development and is crucial for the maintenance of the differentiated status of luminal epithelial cells. The role of GATA3 in breast cancer as a tumor suppressor has been established, although insights into the mechanism of GATA3 expression loss are still required.

**Methods:**

Chromatin immunoprecipitation assays were conducted to study progestin modulation of recruitment of transcription factors to GATA3 promoter. We performed western blot and reverse RT-qPCR experiments to explore progestin regulation of GATA3 protein and mRNA expression respectively. Confocal microscopy and *in vitro* phosphorylation studies were conducted to examine progestin capacity to induce GATA3 serine phosphorylation in its 308 residue. GATA3 participation in progestin-induced breast cancer growth was addressed in *in vitro* proliferation and *in vivo* tumor growth experiments.

**Results:**

In this study, we demonstrate that progestin-activated progesterone receptor (PR) reduces GATA3 expression through regulation at the transcriptional and post-translational levels in breast cancer cells. In the former mechanism, the histone methyltransferase enhancer of zeste homolog 2 is co-recruited with activated PR to a putative progesterone response element in the *GATA3* proximal promoter, increasing H3K27me3 levels and inducing chromatin compaction, resulting in decreased GATA3 mRNA levels. This transcriptional regulation is coupled with increased GATA3 protein turnover through progestin-induced GATA3 phosphorylation at serine 308 followed by 26S proteasome-mediated degradation. Both molecular mechanisms converge to accomplish decreased GATA3 expression levels in breast cancer cells upon PR activation. In addition, we demonstrated that decreased GATA3 levels are required for progestin-induced upregulation of cyclin A2, which mediates the G1 to S phase transition of the cell cycle and was reported to be associated with poor prognosis in breast cancer. Finally, we showed that downregulation of GATA3 is required for progestin stimulation of both *in vitro* cell proliferation and *in vivo* tumor growth.

**Conclusions:**

In the present study, we reveal that progestin-induced PR activation leads to loss of GATA3 expression in breast cancer cells through transcriptional and post-translational regulation. Importantly, we demonstrate that GATA3 downregulation is required for progestin-induced upregulation of cyclin A2 and for progestin-induced *in vitro* and *in vivo* breast cancer cell growth.

**Electronic supplementary material:**

The online version of this article (doi:10.1186/s13058-014-0491-x) contains supplementary material, which is available to authorized users.

## Introduction

Progesterone is an ovarian steroid hormone essential for breast development and implicated in breast cancer progression. Progesterone receptors (PR) exist primarily as two coexpressed isoforms, PR-A and PR-B [1] encoded in the same gene downstream of distinct promoters [2]. Ligand binding triggers a network of signaling events which targets PR and the protein components of chromatin resulting in chromatin remodeling for access of transcription factors to DNA [3]. Nuclear PR, together with coregulators, activates or represses transcription of PR target genes, either directly through DNA binding to progesterone response elements (PRE) or indirectly through tethering interactions with other transcription factors [4]-[7]. Recent genome-wide studies showed that 63% of the PR binding sites associated with transcriptional gene regulation contain PREs within PR binding sites [8]. However, the lack of strong PREs in other PR binding sites highlights the relevance of additional regulatory mechanisms such as tethering, pioneer transcription factor binding and PR cofactor recruitment, which may account for cell-specific PR cistromes [8].

On the other hand, the GATA family of transcription factors is involved in cell fate determination [9]. It has been demonstrated that GATA3 is a critical regulator in both mouse and human development, since constitutive null mutations of GATA3 result in embryonic lethality [10]. Recent evidence identified GATA3 as the most highly expressed transcription factor in the terminal end buds of the mammary gland during development [11]. In addition, GATA3 expression is necessary for specification and maintenance of both ductal and alveolar luminal cell fate [11],[12]. The requirement of GATA3 for the development of the mammary gland and for the maintenance of the luminal cell fate suggested potential implications for GATA3 in the breast cancer scenario. Using the mouse mammary tumor virus LTR-driven polyoma middle T antigen (MMTV-PyMT) mouse model of tumor progression, Kouros-Mehr *et al.* demonstrated that GATA3 is downregulated in carcinomas compared to adenomas, and that GATA3 is the only member of the GATA family of transcription factors which is differentially expressed between these two subsets [13]. Importantly, it was demonstrated that the loss of GATA3 expression marks the loss of tumor differentiation and the onset of tumor dissemination [13]. Remarkably, reconstitution of GATA3 expression by retroviral transduction of MMTV-PyMT mice tumor outgrowths proved to be sufficient to suppress tumor dissemination. In addition, restoration of GATA3 induced differentiation of mammary ductal adenocarcinomas [13]. As demonstrated by a transgenic mouse model overexpressing GATA3, increased expression of GATA3 delays tumor growth, reduces tumor-initiating capacity and increases tumor differentiation [14]. Collectively, these findings highlight the capability of GATA3 to induce functional differentiation of mammary ductal adenocarcinomas and reduction of the tumor initiating capacity in transplanted tumor cells. However, silencing of the *GATA3* locus is not sufficient to promote malignant progression, since premature loss of GATA3 expression in well-differentiated adenomas promotes widespread detachment of cells from the basement membrane followed by apoptosis [13]. In breast cancer patients, GATA3 expression displays a steady decrease with increasing tumor grade [15], and several independent studies demonstrated that GATA3 expression holds independent prognostic value, with increased GATA3 expression correlating with good prognosis [15]-[19]. A recent study identified *GATA3* as one of the only three genes carrying somatic mutations with more than 10% incidence across breast cancer subtypes defined by the mRNA expression profile [20]. Taken together, these studies highlight the role of GATA3 as a tumor suppressor.

A functional link between GATA3 expression and the histone methyltransferase enhancer of zeste homolog 2 (EZH2) has been suggested, since EZH2 knockdown in basal-like breast cancer cells promotes the increase of GATA3 expression levels [21]. EZH2 is the catalytic subunit of the Polycomb Repressor Complex 2 (PRC2), and catalyzes the tri-methylation of lysine 27 of histone H3 (H3K27me3), a histone mark associated with chromatin compaction and transcriptional repression [22],[23]. Notably, Polycomb group (PcG) target genes are mainly involved in embryonic development and cell differentiation [24]. During pregnancy, EZH2 expression levels, phosphorylation of EZH2 at threonine 487 (pT487-EZH2) and total H3K27me3 are increased in the mammary gland, correlating with increased progesterone levels and PR expression. Moreover, PR knockdown results in decreased pT487-EZH2 in the human breast cancer cell line T47D [25]. Overexpression of EZH2 has been detected in breast cancer, with increased EZH2 levels correlating with higher proliferation rates, neoplastic transformation and more aggressive cancer subtypes [26],[27].

On the other hand, cyclin A has a role in mediating the transition of G1 to S and G2 to M phase of the cell cycle, since it is required for the onset of DNA replication and mitosis [28],[29]. While expression of cyclin A1 is limited to meiosis of germinal cells and early embryos, cyclin A2 is expressed in proliferating somatic cells. Potential implications of cyclin A2 in breast cancer have been suggested, since transgenic mice overexpressing cyclin A2 generate aberrant nuclear abnormalities suggestive of preneoplasic alterations [30]. In addition, cyclin A2 expression levels have been shown to have independent prognostic value in breast cancer patients, with increased expression correlating with poor outcome [31]. Accordingly, cyclin A2 was found to be significantly downregulated in MDA-MB-231 breast cancer cells engineered to overexpress GATA3 [32].

In the present work, we demonstrated that progestin-induced PR activation promotes the loss of GATA3 expression in breast cancer cells through transcriptional and post-translational regulation. Finally, we have also shown that GATA3 downregulation is required for progestin-induced *in vitro* cell proliferation and *in vivo* breast tumor growth.

## Methods

### Animals and tumors

Experiments were carried out using female BALB/c mice raised at the Instituto de Biología y Medicina Experimental (IBYME), Buenos Aires, Argentina. Animal experiments were performed as described previously [33] in accordance with the highest standards of animal care, as outlined in the US National Institutes of Health *Guide for the Care and Use of Laboratory Animals* [34]. The experiments were approved by the IBYME Animals Research Committee [5]. The C4HD tumor line displays high levels of estrogen receptor (ER) and PR. This tumor line does not express glucocorticoid or androgen receptors. Progestins exert a sustained proliferative response *in vitro* and *in vivo* in the C4HD tumor model [35].

### Reagents

Medroxyprogesterone acetate (MPA), RU486, (Dulbecco’s) Modified Eagle’s Medium: Ham F12 1:1 ((D)MEM) and progesterone were purchased from Sigma Aldrich (Saint Luis, MO, USA). The transfection reagents Fugene HD and XtremeGENE HP (Roche Biochemicals, Indianapolis, IN, USA) were used according to the manufacturer’s instructions. Cycloheximide was from Sigma-Aldrich (St Louis, MO, USA) and was used at 1 μM final concentration. Bortezomib (VELCADE®) was from Millennium Pharmaceuticals, Inc (Cambridge, MA, USA) and was used at 100 nM final concentration; actinomycin D was from Sigma-Aldrich (St Louis, MO, USA) and was used at a final concentration of 5 μg/ml; myristoylated cAMP-dependent protein kinase inhibitor (PKI) was from Enzo Life Sciences (Exeter, UK) and was used at 1 μM final concentration.

### Antibodies

The following antibodies were used: anti-GATA3 (HG3-31, sc-268), anti-cyclin A (C-19, sc-596), anti-cyclin E (HE12, sc-247), anti-ER (MC-20, sc-542) and anti-PR (H-190, sc-7208), from Santa Cruz Biotechnology (Santa Cruz, CA, USA); anti-pSer308-GATA3 (ab61052), anti-Histone H3 (tri-methyl K27) (ab6002) and anti-Histone H3 (acetyl K9) (ab4441) from Abcam (Cambridge, MA, USA); anti-PR (Ab7), anti-actin (clone ACTN05) and anti-cyclin D1 (RB 9041-P1) from Neomarkers (Freemont, CA, USA); anti-GAPDH (D16H11), anti-phospho-PKA Substrate (RRXS*/T*)(100G7E) and anti-PKA C-α (4782) from Cell Signaling (Beverly, MA, USA); anti-EZH2 (#39933) from Active Motif (Carlsbad, CA,USA); anti-acetyl-Histone H4 (#06-866) from Millipore (Temecula, CA, USA); and anti-β-tubulin from Sigma.

### Cell culture, treatments and proliferation assays

The human breast cancer cell line T47D was obtained from the American Type Culture Collection (ATCC) (Manassas, VA, USA) and T47D-Y cells were a generous gift from Dr. Horwitz (University of Colorado Health Sciences Center, Denver, CO, USA). Both cell lines were maintained in (D)MEM supplemented with 10% fetal calf serum (FCS). Proliferation assays and treatments were performed in (D)MEM for the T47D cell line. The human breast cancer cell line BT474 was purchased from the ATCC and maintained in Roswell Park Memorial Institute (RPMI) 10% FCS. Primary cultures of epithelial cells from C4HD tumors were performed as previously described [5]. C4HD cells were starved and treated in (D)MEM supplemented with 0.1% of FCS previously depleted of steroids by treatment with active charcoal (chFCS). For proliferation assays, 1 x 10^4^ cells were plated in 96-well plates and allowed to attach overnight. Cells were starved in (D)MEM for T47D cells or in (D)MEM 0.1% chFCS for C4HD cells or (D)MEM 1% chFCS for BT474 cells. Treatments were performed in (D)MEM or (D)MEM 0.1% chFCS, respectively, with 10 nM MPA or control vehicle (1:1000 ethanol) for 24 hours. Cell proliferation was evaluated as the incorporation of 1 μCi [^3^H]-thymidine during the last 18 hours of incubation (New England Nuclear, DuPont, Boston, MA, USA; specific activity 20 Ci/mmol) as previously described [36]. Assays were performed in octuplicate.

### Western blots

Lysates were prepared from cells subjected to the different treatments described in each experiment and 50 μg of proteins were resolved by SDS-page as previously described [33]. Membranes were immunoblotted with the antibodies described in each experiment.

### Chromatin immunoprecipitation

Chromatin immunoprecipitation assays were performed as previously described [6]. Primers used for q-PCR are listed in Table S1 (see Additional file 1).

### RNA preparation and real-time RT-PCR

Total RNA was isolated from T47D or C4HD cells treated as indicated by using TRIzol reagent (Invitrogen, Carlsbad, CA, USA) according to the manufacturer’s protocol. One microgram of RNA was reverse transcribed using Superscript III reverse transcriptase (RT) (Invitrogen) according to the manufacturer’s instructions. Primer sequences are detailed in Table S1 (see Additional file 1). PCR was performed for 40 cycles with 15 seconds of denaturing at 95°C and annealing and extension at 60°C for one minute. The fold change of mRNA expression was calculated by normalizing the absolute GATA3 mRNA amounts to GAPDH mRNA levels used as an internal control.

### DNAse sensitivity assay

Cells were harvested in lysis buffer (10 mM Tris-Cl, pH 7.5; 10 mM NaCl; 3 mM MgCl2; 0.05% NP40) and incubated on ice for 10 minutes. Nuclei were isolated by centrifugation at 2,000 rpm for five minutes at 4°C. The pellet was washed with digestion buffer (40 mM Tris-Cl pH 7.5; 10 mM MgCl2; 1 mM CaCl2) and centrifuged at 2,000 rpm for five minutes at 4°C. Pellets were resuspended in 200 μl and each treatment was divided in two tubes (cut and uncut as input control). Samples were incubated or not with DNAse I (QR1) (Promega Madison, WI, USA) for five minutes at 37°C. The reaction was stopped by adding 50 μg of proteinase K and incubating at 65°C for two hours. DNA was isolated by phenol extraction and ethanol precipitation. Sensitivity was measured by performing q-PCR of 100 ng of DNA and calculated as 2^^(Ctcut – Ctuncut)^.

### *In vivo*tumor growth

C4HD cells were transiently transfected with the vectors detailed in the results section. After transfection, 1 x 10^6^ cells were inoculated subcutaneously (s.c.) into BALB/c mice treated with a 40-mg MPA depot in the flank opposite to cell inoculum. Tumor volume was calculated as previously described. The area under the curve for each sample of each experimental group was plotted and one-way analysis of variance (ANOVA) plus Bonferroni post-test were applied to assess statistical significance of differences in tumor growth between groups.

### Plasmids

The pEGFP-N1 empty vector was obtained from BD Biosciences-Clontech (Palo Alto, CA, USA). The expression vector encoding GATA3 as a fusion protein with GFP within the pEGFP-C1 backbone was a generous gift from Dr. Nakayama (Department of Immunology, Graduate School of Medicine, Chiba University, Japan). Site directed mutagenesis was performed on the pEGFP-C1-GATA3 to produce the replacement of serine 308 for alanine, using the primers detailed in Table S1 (see Additional file 1). Successful mutation of the sequence was verified by DNA sequencing at the Instituto Nacional de Tecnología Agropecuaria (INTA) (Buenos Aires, Argentina). The plasmids encoding human wild-type PR-B [37] and wild-type PR-A [38] were kindly provided by Dr. Horwitz and Dr. Edwards (Baylor College of Medicine, Houston, TX, USA), respectively.

### siRNA transfections

siRNAs targeting mouse PR were synthesized by SAB Biosciences (Valencia, CA, USA) and siRNAs targeting human PR were synthesized by Dharmacon (Lafayette, CO, USA). A siRNA which does not target any known mammalian gene was used as a negative control. The transfection of siRNA duplexes was performed for two days using DharmaFECT (Dharmacon) transfection reagent according to the manufacturer’s instructions. All siRNA sequences are stated in Table S2 (see Additional file 1).

### *In vitro*GATA3 phosphorylation

To study the capacity of endogenous protein kinase (PKA) to phosphorylate GATA3, T47D cells were treated with MPA for eight hours, pretreated with PKI for 30 minutes prior to MPA treatment or kept untreated. Cells were lysed and PKAs were immunoprecipitated from 1.5 mg of protein extracts from each cell treatment with an anti-PKA antibody. GATA3 was immunoprecipitated from 0.5 mg of protein from T47D cells growing in (D)MEM. Immunoprecipitated GATA3 was subjected to an *in vitro* phosphorylation assay with PKAs immunoprecipitated from cells subjected to each of the treatments described above and as described in [39]. Samples were analyzed by SDS-PAGE.

### Flow cytometry

T47D cells treated with MPA for the indicated times were harvested with trypsin and fixed in PBS 1% paraformaldehyde. To determine GATA3 serine phosphorylation, cells were permeabilized with permeabilization buffer (PB) (PBS 10% FCS 0.5% saponine) and labeled with anti-pSer308-GATA3 (1 μg of anti-pSer308-GATA3 antibody per 1 x 10^6^ cells) followed by incubation with anti-rabbit IgG-Alexa 488. Background staining was evaluated in cells incubated with an isotype control immunoglobulin G (IgG) followed by anti-rabbit IgG Alexa 488-conjugated antibody. Cells were washed twice with PB, followed by RNA digestion (RNAse A 50 U/ml) and propidium iodide (20 μg/ml) staining for 30 minutes at room temperature in the dark. Cell cycle analysis by propidium iodide content and determination of phospho-GATA3 expression by Alexa 488 fluorescence, were measured using a Canto II flow cytometer (Becton–Dickinson, La Jolla, CA, USA). The acquired data were analyzed by FlowJo v5.7.2 software. Delta mean fluorescence intensity (MFI) values were obtained by subtracting the MFI of the cells incubated with control isotype antibody from the MFI of cells incubated with anti-pSer308-GATA3 antibody.

### Immunohistochemistry

Immunohistochemistry was performed as previously described [40]. Briefly, antigen retrieval was performed in 10 mM sodium citrate buffer pH 6 for 20 minutes at 96°C to 98°C. Slides were incubated with the primary antibodies indicated in the Antibodies section. Sections were subsequently incubated with the polydetector horseradish peroxidase (HRP) system (Bio SB, Santa Barbara, CA, USA) and developed in 3-3′-diaminobenzidine tetrahydrochloride.

### Immunofluorescence and confocal microscopy

Cells grown in glass coverslips were fixed and permeabilized with ice-cold methanol and were then blocked in PBS 1% bovine serum albumin (BSA). pSer308-GATA3 was localized using the anti-pSer308-GATA3 antibody described previously, followed by incubation with anti-rabbit IgG-Alexa 488 (Molecular Probes, Eugene, OR, USA). Negative controls were carried out using PBS instead of primary antibodies. Nuclei were detected by propidium iodide staining (5 μg/ml). Cells were analyzed using a Nikon Eclipse E800 confocal laser microscopy system.

## Results

### Progesterone receptor activation promotes downregulation of GATA3 protein expression

**Figure 1:**
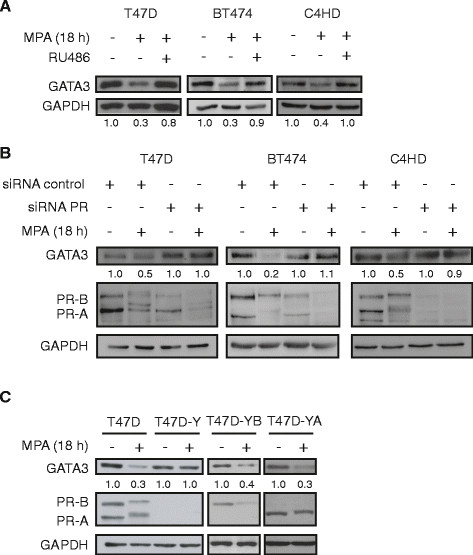
**PR activation promotes GATA3 downregulation. A)** T47D, BT474 or C4HD cells were pretreated or not with the antiprogestin RU486 (10 nM) for 30 minutes and then coincubated with MPA (10 nM) for the indicated time. Total protein lysates were prepared and GATA3 expression was measured by western blot (WB). GATA3 bands underwent densitometry and values were normalized to GAPDH protein bands setting the value of untreated cells as 1.0. **B)** T47D, BT474 or C4HD cells were transfected with control siRNA or with siRNA targeting PR (siRNA PR). Cells were starved for 24 hours and treated with MPA for the indicated time, total protein lysates were prepared and GATA3 expression was measured by WB. Bands were quantified as described in panel A. **C)** T47D cells, PR-null T47D-Y cells or T47D-Y cells transfected with either PR-A (T47D-YA) or PR-B (T47D-YB) isoforms were treated with MPA for 18 hours. Bands were quantified as described in panel A. MPA, medroxyprogesterone acetate; PR, progesterone receptor.

In the present work we used primary cultures of C4HD epithelial cells from a model of mammary carcinogenesis induced by MPA in female BALB/c mice [33] and the PR-positive human breast cancer cell lines T47D and BT474 [41]. Our first aim was to evaluate whether GATA3 was regulated by PR activation. We found that MPA treatment caused GATA3 downregulation in T47D, BT474 and C4HD cells (Figure 1A). This effect was prevented by preincubation with the antiprogestin RU486 in all cell lines tested (Figure 1A) showing the requirement of the activation of the classical PR for GATA3 downregulation. T47D cells treated with increasing concentrations of progesterone, the endogenous PR ligand, displayed similar levels of GATA3 downregulation to MPA-treated cells (see Additional file 1: Figure S1). To further demonstrate the requirement of PR for progestin-mediated GATA3 downregulation, we silenced PR expression using siRNAs and then performed MPA treatment. In line with the previous results, PR knockdown prevented GATA3 downregulation by MPA in T47D, BT474 and C4HD cells (Figure 1B). Accordingly, in the PR-null T47D-Y cells, which derive from the T47D cell line, MPA failed to regulate GATA3 expression (Figure 1C). Reconstitution of either isoform B (PR-B) or isoform A (PR-A) of the PR restored GATA3 regulation by MPA treatment (Figure 1C) indicating that both isoforms are capable of mediating the MPA effect on GATA3 expression. These results demonstrate a PR-mediated decrease of GATA3 expression in breast cancer cells upon progestin treatment.

### Progestins induce transcriptional repression of GATA3 gene by co-recruitment of progesterone receptor and EZH2

**Figure 2:**
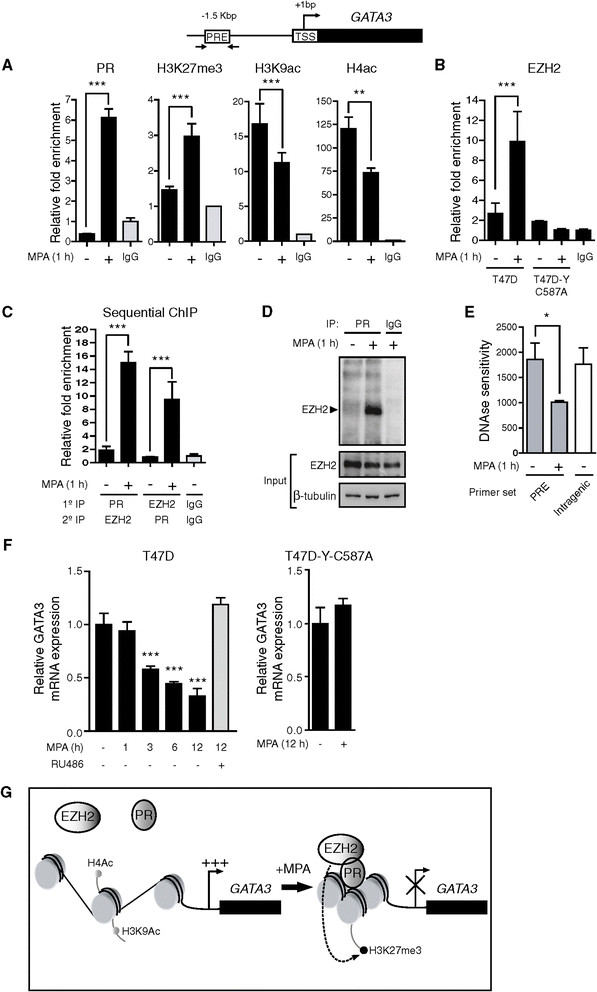
**Transcriptional repression of GATA3 gene by co-recruitment of PR and EZH2. A)** Protein recruitment to the *GATA3* promoter was analyzed by ChIP in cells treated as indicated. Immunoprecipitated DNA was amplified by q-PCR using primers flanking the potential PRE located at position −1,504 bp. Each sample was normalized to the input. Data are expressed as n-fold chromatin enrichment over isotype control. **B)** T47D or T47D-Y-C587A cells were treated and ChIP was performed as described in A. **C)** Chromatins from T47D cells treated or not with MPA were first immunoprecipitated with PR antibody and then re-immunoprecipitated using EZH2 antibody. The inverse immunoprecipitation order is also shown. q-PCR analysis of immunoprecipitated DNA was performed as detailed in A. **D)** Nuclear extracts from T47D cells treated as indicated were immunoprecipitated (IP) with PR antibody and analyzed by WB with EZH2 antibody . As control of specificity, lysates were immunoprecipitated with an isotype control. Input cell lysates were blotted in parallel. **E)** A DNAse sensitivity assay was performed as described in the Methods section, using the indicated primers detailed in Additional file 1, Table S1. **F)** T47D or T47D-Y-C587A cells were treated as indicated and GATA3 mRNA expression levels were determined by RT-qPCR. The fold change of mRNA expression levels upon MPA treatment for the indicated times was calculated by normalizing the absolute levels of GATA3 mRNA to GAPDH levels, which were used as an internal control, and setting the value of untreated cells as 1.0. For A, B, C and F, (*P <0.05, **P <0.01 and ***P <0.001, one-way ANOVA). **G)** Schematic representation of the mechanism proposed for progestin-induced GATA3 transcriptional repression. ANOVA, analysis of variance; ChIP, chromatin immunoprecipitation; EZH2, enhancer of zeste homolog 2; MPA, medroxyprogesterone acetate; PR, progesterone receptor; PRE, progesterone response element; WB, Western blot.

We next explored the mechanism of GATA3 downregulation at the transcriptional level. For this purpose, we performed an *in silico* search [42] for PREs within 5 kb upstream of the transcriptional start site (TSS) of *GATA3* and found a putative PRE located at position −1,504 bp to −1,498 bp relative to the TSS (see Additional file 1: Figure S2). By performing chromatin immunoprecipitation (ChIP) assays we found that PR was significantly recruited to the potential PRE site in the presence of MPA both in T47D (Figure 2A) and in BT474 cells (see Additional file 1: Figure S3). We also found a concomitant increase in H3K27me3 (Figure 2A and Additional file 1: Figure S3), a histone modification associated with transcriptional repression [22],[23]. Consistently, we observed a decrease in the acetylation of lysine 9 of histone H3 (H3K9ac) and in the total acetylation of histone H4 (H4ac) (Figure 2A). Since H3K27me3 is catalyzed by the histone methyltransferase EZH2, we tested progestin regulation of EZH2 binding to the putative PRE site located in *GATA3* promoter. Indeed, EZH2 was significantly recruited to this site in MPA-treated T47D cells (Figure 2B). To study the requirement of PR binding to DNA for EZH2 recruitment, we used the T47D-Y-C587A cell line, which stably expresses a PR harboring a substitution of the cysteine 587 for alanine that renders the receptor unable to bind to DNA or to tether to other transcription factors bound to DNA [43]. Progestin treatment of T47D-Y-C587A failed to induce EZH2 recruitment to the putative PRE (Figure 2B), indicating the requirement of PR binding to this site in order to enable EZH2 recruitment upstream of the *GATA3* gene. In order to further support progestin-induced simultaneous PR and EZH2 binding to DNA, we performed sequential ChIP experiments and found that progestin-treated cells display a significantly increased co-recruitment of PR and EZH2 to the potential PRE as compared to control cells (Figure 2C). Our results on PR and EZH2 nuclear interaction were reinforced by coimmunoprecipitation experiments, showing an increase in PR and EZH2 interaction in MPA-treated cells (Figure 2D). Since H3K27me3 is associated with transcriptional repression by chromatin compaction, we next assessed chromatin accessibility at the PRE by performing DNAse I sensitivity assays using an intragenic region of *GATA3* as sensitivity control. We found that MPA treatment decreases DNAse I sensitivity, consistent with a less accessible and repressive chromatin environment both at the potential PRE and at the TSS regions (Figure 2E and Additional file 1: Figure S4). In line with these results, a decrease in GATA3 mRNA levels is observed upon MPA treatment, while RU486 pretreatment prevented this regulation in both T47D and C4HD cells (Figure 2F; Additional file 1: Figure S5). GATA3 mRNA was not downregulated in progestin-treated T47D-Y-C587A cells (Figure 2F, right panel), which is in accordance with the lack of EZH2 recruitment observed in Figure 2B. Taken together, these results demonstrate a PR-mediated *GATA3* transcriptional repression through co-recruitment of PR and EZH2 to a putative PRE site at the proximal region of *GATA3* promoter, with the concomitant increase of H3K27me3 and decrease of chromatin accessibility, as depicted in Figure 2G.

### Progestin exerts GATA3 post-translational regulation through the 26S proteasome

**Figure 3:**
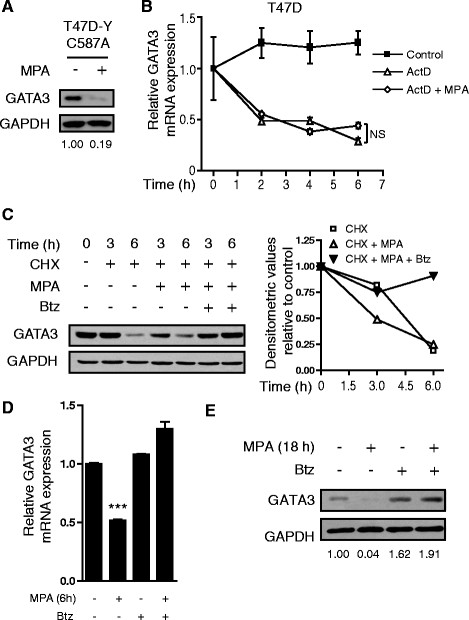
**Post-translational regulation of GATA3 through the 26S proteasome. A)** T47D-Y-C587A cells were treated with MPA for 18 hours, protein cell lysates were prepared and immunoblotted for the indicated proteins. Signal intensities of GATA3 bands in the WBs were analyzed by densitometry, values were normalized to GAPDH protein bands setting the value of untreated cells as 1.0. **B)** T47D cells were treated or not with MPA and/or actinomycin D (5 μg/μl) for the indicated times. The fold change of mRNA expression levels upon MPA treatment for the indicated times was calculated by normalizing the absolute levels of GATA3 mRNA to GAPDH levels, which were used as an internal control, and setting the value of untreated cells as 1.0. Not significant (NS), one-way ANOVA. **C)** T47D cells were treated or not with cycloheximide (CHX) at 1 μM final concentration and/or with MPA or bortezomib (Btz) at 100 nM final concentration as indicated, protein cell lysates were prepared and GATA3 expression was measured by WB. Signal intensities of GATA3 bands in the WBs were analyzed as indicated in A. **D)** T47D cells were pretreated or not with Btz for 30 minutes and then treated or not with MPA as indicated, and GATA3 mRNA expression was measured by RT-qPCR as described for B (***P <0.001, one-way ANOVA). **E)** T47D cells were treated or not with MPA and/or Btz as indicated, protein cell lysates were prepared and GATA3 expression was measured by WB. Signal intensities of GATA3 bands in the WBs were analyzed as indicated in A. ANOVA, analysis of variance; MPA, medroxyprogesterone acetate; WB, Western blot.

We found that despite MPA inability to mediate GATA3 transcriptional repression in T47D-Y-C587A cells (Figure 2F) total GATA3 protein levels were still downregulated by MPA in this cell line (Figure 3A). This result indicates the existence of an additional level of regulation of GATA3 expression.

Since it has been reported that GATA3 mRNA stability is regulated by RNA binding proteins [44], we first studied the capacity of MPA to regulate the stability of GATA3 mRNA. For this purpose we used the transcription inhibitor actinomycin D (ActD) and measured the stability of GATA3 mRNA with or without the addition of MPA. No significant changes were detected in ActD-treated cells upon progestin treatment (Figure 3B), ruling out MPA regulation of GATA3 mRNA stability.

Next, we analyzed GATA3 protein stability using the translation inhibitor cycloheximide (CHX). Inhibition of translation with CHX allowed us to measure the stability of the remaining GATA3 protein relative to initial GATA3 levels. In accordance with previous reports [45], translation impairment by CHX promoted GATA3 protein decay within six hours. We found that MPA treatment increased GATA3 turnover (Figure 3C; lane 2 versus lane 4, the densitometric analysis is shown in the right panel). Since previous studies have shown GATA3 26S proteasome-mediated degradation in immune system cells [46], we hypothesized that the 26S proteasome could be involved in MPA-mediated GATA3 degradation in breast cancer cells. In order to address whether GATA3 stability was regulated in a 26S proteasome-dependent manner, we treated cells with CHX in combination with the 26S proteasome pharmacological inhibitor bortezomib (Btz). Impairment of the 26S proteasome function resulted in prevention of GATA3 protein degradation in the presence of progestin (Figure 3C; lanes 4 and 5 versus lanes 6 and 7). This result demonstrates that the 26S proteasome activity is required for the decrease of GATA3 protein levels by MPA. Previous studies have shown that PR transcriptional activity depends on the 26S proteasome function [47]. Therefore, we speculated that PR-mediated GATA3 repression could be prevented both at the transcriptional and post-translational levels by inhibiting the 26S proteasome activity. Indeed, incubation with Btz resulted in prevention of GATA3 downregulation upon MPA treatment both at the mRNA (Figure 3D) and protein levels in T47D and C4HD cells (Figure 3E and Additional file 1: Figure S6). These results demonstrate, on the one hand, that GATA3 protein levels are regulated by progestin in a post-translational manner and, on the other, that inhibition of the 26S proteasome activity prevents MPA-mediated GATA3 downregulation at both transcriptional and post-translational levels.

### GATA3 phosphorylation at serine 308 regulates protein stability

**Figure 4:**
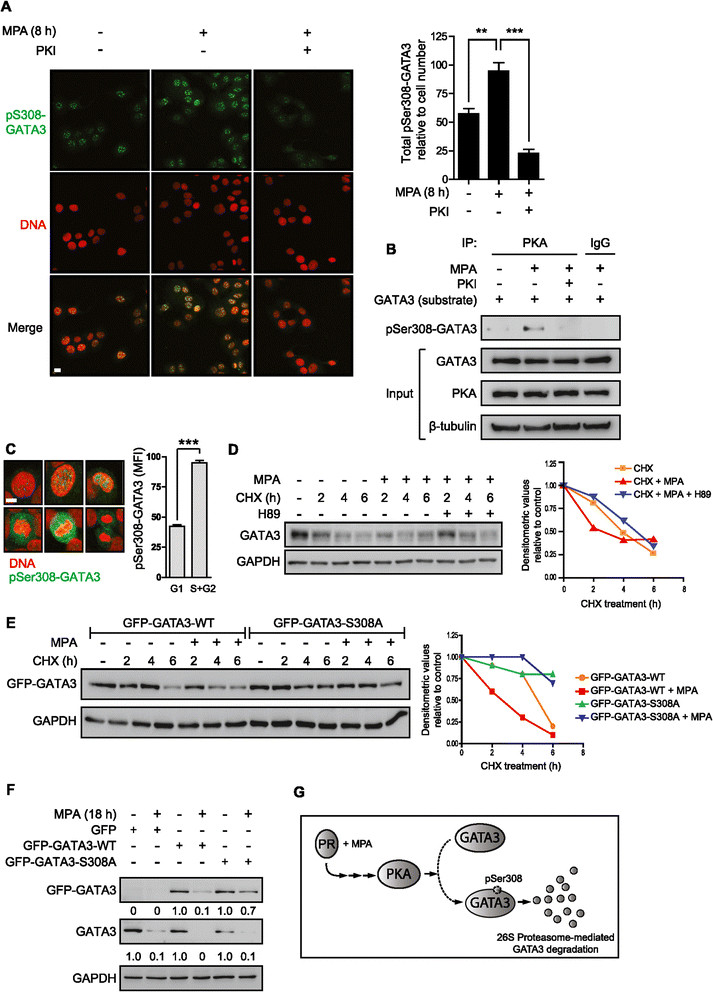
**GATA3 phosphorylation regulates protein stability. A)** T47D cells were treated or not with MPA and the PKA inhibitor PKI (1 μM) for eight hours. Nuclei were stained with propidium iodide and confocal microscopy analysis was performed. Scale bar = 10 μm. Right panel: Quantification of pSer308-GATA3 positive cells for the treatments performed in A. Total pSer308-GATA3 intensity relative to cell number was determined for a minimum of 150 cells per treatment (N = 6) (**P <0.01, ***P <0.001, one-way ANOVA). **B)** PKA induces pSer308-GATA3 *in vitro*. T47D cells treated as indicated were lysed and PKAs were immunoprecipitated from protein extracts from each cell treatment. GATA3 was immunoprecipitated from T47D cells growing in (D)MEM. Immunoprecipitated GATA3 was subjected to a cold *in vitro* phoshorylation assay as described in the Methods section. **C)** Confocal microscopy images of pSer308-GATA3 of cells in different stages of the cell cycle as determined by propidium iodide staining. Scale bar = 10 μm. The bar plot on the right panel represents a flow cytometry analysis of pSer308-GATA3 mean fluorescence intensity (MFI) of different stages of the cell cycle as determined by DNA content (***P <0.001, Student’s *t*-test). **D)** T47D cells were treated with CHX and/or with MPA or H89 (1 μM) for the indicated times. GATA3 bands of WBs underwent densitometry and values were normalized to GAPDH protein bands setting the value of untreated cells as 1.0. The results are graphically represented in a line plot. **E)** Cells were transfected with the indicated vectors for 48 hours, starved for 24 hours and treated as indicated. Densitometric analysis of WB was performed as in D. **F)** Cells were transfected as in E and densitometric analysis was performed as in D. **G)** Proposed model for GATA3 post-translational regulation by progestin. ANOVA, analysis of variance; CHX, cycloheximide; MPA, medroxyprogesterone acetate; PKA, protein kinase; WB, Western blot.

We next addressed whether MPA induces GATA3 phosphorylation at serine 308 (pSer308-GATA3), which is located within a cAMP-dependent PKA consensus site and has been shown to be a PKA target *in vitro* [48]. Confocal microscopy experiments revealed an increase in phosphorylated PKA substrates in T47D cells treated with MPA and that incubation with the myristoylated PKA inhibitor (PKI) prevented this effect (see Additional file 1: Figure S7). An increase in pSer308-GATA3 positive cells upon progestin treatment was demonstrated by incubation with an anti-pSer308-GATA3 antibody followed by incubation with an Alexa 488-conjugated secondary antibody (Figure 4A). Specificity of the pSer308-GATA3 antibody was verified by colocalization with total GATA3 and loss of signal by GATA3 knockdown (see Additional file 1: Figure S8). MPA and PKI coincubation prevented MPA-induced GATA3 phosphorylation, as demonstrated by the decrease in pSer308-GATA3 positive cells (Figure 4A right panel), showing the requirement of PKA activation for GATA3 phosphorylation at serine 308 by MPA. To directly assess whether PKA could induce phosphorylation of pSer308-GATA3, immunoprecipitated GATA3 was subjected to a cold *in vitro* phosphorylation assay as described in the Methods section. MPA-activated PKA induced pSer308-GATA3, which was blocked when cells were pretreated with PKI (Figure 4B).

On the other hand, PR downregulation by the 26S proteasome is required for its transcriptional hyperactivity [49]. To demonstrate that inhibition of the 26S proteasome blocked GATA3 degradation due to the effect of the proteasome *per se* on GATA3 and not due to impairment of MPA-induced GATA3 phosphorylation, we evaluated the effect of Btz on GATA3 phosphorylation. Confocal microscopy assays showed that pSer308-GATA3 is induced in the presence of Btz indicating that the 26S proteasome blockade does not affect the MPA-induced increase of pSer308-GATA3 (see Additional file 1: Figure S9).

Notably, cells positive for pSer308-GATA3 were undergoing mitosis, as evidenced by chromatin staining with propidium iodide (Figure 4C). This observation was confirmed by flow cytometry analysis, showing increased pSer308-GATA3 levels in MPA-treated cells in the S and G2 phase of the cell cycle compared to cells in G1 (Figure 4C, right panel). MPA-induced pSer308-GATA3 was also observed as a function of DNA content (see Additional file 1: Figure S10). We next addressed the relevance of GATA3 phosphorylation at serine 308 for GATA3 protein stability. For this purpose, we treated T47D cells with the PKA inhibitor H89 and measured GATA3 protein turnover by treatment with CHX in the presence or absence of MPA. Densitometric analysis showed that PKA inhibition restored GATA3 protein stability to levels similar to those of cells treated with CHX alone (Figure 4D; the densitometric analysis is shown in the right panel). To further support the importance of GATA3 phosphorylation at serine 308 for protein stability, we generated a serine-to-alanine substitution in position 308 (GFP-GATA3-S308A), a non-phosphorylable amino acid, in a vector encoding a GFP-fused wild type protein (GFP-GATA3-WT). Here, we show by WB analysis that cells transfected with GFP-GATA3-S308A and treated with CHX showed increased stability of the GFP-GATA3-S308A protein compared to its wild type counterpart, even in the presence of the progestin (Figure 4E; the densitometric analysis is shown in the right panel). Furthermore, transfection experiments revealed that although MPA treatment promoted downregulation of both endogenous GATA3 and GFP-GATA3-WT proteins, it was unable to downregulate the GFP-GATA3-S308A mutant protein to a similar extent (Figure 4F). Since a constitutive promoter drives exogenous GATA3 expression, this experiment validates, on the one hand, the MPA-mediated post-translational downregulation of GATA3 and, on the other hand, demonstrates the requirement of phosphorylation at serine 308 for increased GATA3 protein turnover. The presented results link GATA3 phosphorylation at serine 308 with protein stability and demonstrate the requirement of GATA3 phosphorylation to achieve MPA-induced GATA3 protein degradation. A schematic representation of the mechanism described is shown (Figure 4G).

### GATA3 downregulation is required for progestin-induced cell proliferation and increased cyclin A2 levels

**Figure 5:**
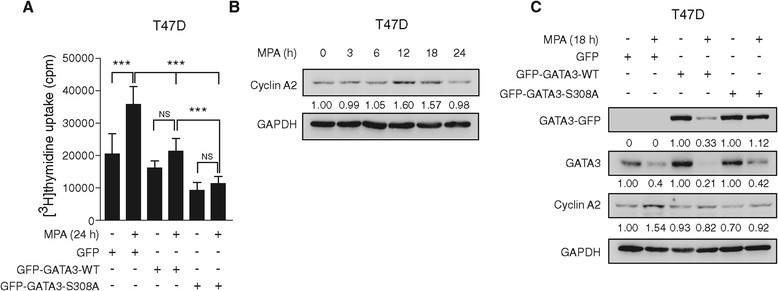
**GATA3 downregulation is required for progestin-induced cell proliferation and increased cyclin A2 levels. A)** T47D cells were transfected with the indicated vectors for 48 hours and cell proliferation was measured as described in the Methods section. Not significant (NS), (***P <0.001, one-way ANOVA). **B)** Cells were treated with MPA for the indicated times, protein lysates were prepared and immunoblotted for the indicated proteins. The densitometry values were obtained by normalizing cyclin A to GAPDH protein bands and setting the value of untreated cells as 1.0. **C)** Cells were transfected with the indicated vectors for 48 hours, starved for 24 hours and treated as indicated. Protein lysates were prepared and immunoblotted for the indicated proteins. Bands were quantified as described for panel B. ANOVA, analysis of variance; MPA, medroxyprogesterone acetate.

Progestin stimulation of *in vitro* and *in vivo* breast cancer is well acknowledged [33],[50]. Given the present results showing that GATA3 is phosphorylated in serine 308 during cell cycle progression (Figure 4C) and that impairment of such phosphorylation prevents GATA3 downregulation by MPA (Figure 4F), we next investigated the association between GATA3 downregulation and progestin-induced *in vitro* proliferation and *in vivo* tumor growth. In order to assess the effect of GATA3 overexpression in cell proliferation, we transfected T47D cells with either GFP-GATA3-WT or GFP-GATA3-S308A and used [^3^H]-thymidine incorporation as a measure of cell proliferation. Transfection of GFP-GATA3-WT vector impaired MPA-induced proliferation in T47D, C4HD and BT474 cells (Figure 5A and Additional file 1: Figure S11, respectively) showing that increased GATA3 levels prevent MPA-induced proliferation. We observed that transfection of the mutant form GFP-GATA3-S308A resulted in a significantly enhanced repression of proliferation compared to GFP-GATA3-WT (Figure 5A). These results are consistent with the previously observed increased stability of the GFP-GATA3-S308A mutant protein in the presence of progestin (Figure 4F) and show the requirement of GATA3 downregulation for MPA-induced cell proliferation.

**Figure 6:**
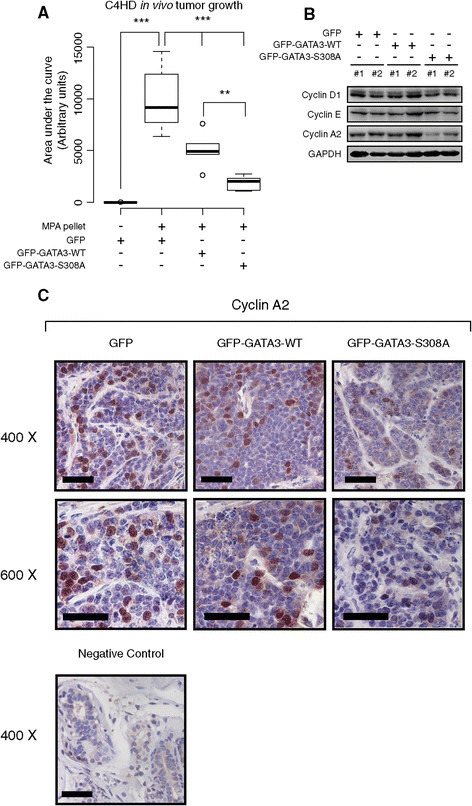
**GATA3 downregulation is required for progestin-induced** ***in vivo*** **tumor growth. A)** C4HD cells (10^6^) from each experimental group were inoculated subcutaneously into female BALB/c mice treated or not with MPA (n = 6 for each experimental group), and tumor volume was calculated using the formula W^2^ x L/2, where W = width and L = length. The boxplot represents the values obtained for the area under the growth curve obtained for each experimental group (***P <0.001 and **P <0.01, one-way ANOVA). **B)** Protein extracts were prepared from tumor samples and immunoblotted for the indicated proteins. **C)** Immunohistochemistry for cyclin A2 in GFP, GFP-GATA3-WT and GFP-GATA3-S308A C4HD tumors. Human normal breast tissue was used as negative control (lower panel). Representative images of each experimental group are shown. Scale bar = 20 μm. ANOVA, analysis of variance; MPA, medroxyprogesterone acetate.

Analysis of a publicly available microarray dataset showed that cyclin A2 was the only member of the cyclin family that was significantly downregulated upon GATA3 overexpression in MDA-MB-231 breast cancer cells [32] (see Additional file 1: Figure S12). We found an increase in cyclin A2 expression levels at 12 hours and 18 hours of progestin treatment (Figure 5B). Moreover, transfection of GFP-GATA-WT or GFP-GATA3-S308A prevented cyclin A2 upregulation in MPA-treated T47D cells (Figure 5C). In line with previous reports [32], MDA-MB-231 cells also displayed decreased cyclin A2 levels when transfected with the GFP-GATA3-WT vector (see Additional file 1: Figure S13). We then addressed the effect of GATA3 overexpression in *in vivo* tumor growth. For this purpose, 10^6^ C4HD cells previously transfected with the GFP control vector, GFP-GATA3-WT, or GFP-GATA3-S308A expression vectors were inoculated subcutaneously into BALB/c mice treated or not with MPA, and tumor growth was measured. C4HD tumor growth displayed a progestin-dependent behavior as previously reported [36], since absence of the MPA pellet resulted in lack of tumor growth in GFP-transfected cells, and inoculation of the MPA pellet induced tumor growth (Figure 6A). GFP-GATA3-WT overexpression significantly reduced progestin-dependent tumor growth, and this effect was significantly enhanced in GFP-GATA3-S308A tumors (Figure 6A). Decreased levels of cyclin A2 were detected in GFP-GATA3-S308A tumors compared to GFP or GATA3-WT tumors, while levels of cyclin D1 and cyclin E were similar across treatments (Figure 6B). The expression levels of cyclin A2 in the tumor samples studied by immunohistochemistry were in line with the results obtained by WB, with decreased cyclin A2 levels in GFP-GATA3-S308A tumors (Figure 6C). Taken together, these results account for a role of GATA3 as a negative regulator of MPA-induced cell proliferation through prevention of cyclin A2 upregulation, and demonstrate the requirement of *GATA3* downregulation for progestin-driven breast cancer growth.

## Discussion

### Progesterone receptor-mediated transcriptional repression

Transcriptional repression is a key step in the regulation of cellular processes. In the present work, we have identified a putative PRE site located in position -1,504 bp relative to the TSS of *GATA3*. We also demonstrated that PR is involved in the transcriptional repression of this gene. We underscored the relevance of *GATA3* repression in PR-induced cell proliferation, and described the capability of PR to co-recruit EZH2, the catalytic subunit of PRC2 to the potential PRE site in *GATA3* proximal promoter. This recruitment resulted in increased H3K27me3, chromatin compaction and transcriptional repression. We have also shown that progestin treatment promotes a decrease in H3K9ac and H4ac, which suggests the involvement of additional mechanisms in the process of *GATA3* repression, such as recruitment of histone deacetylases. Notably, we here show that activation of PR by progestins induces the interaction between PR and EZH2, suggesting that this mechanism could be potentially involved in the repression of other PR target genes. Further investigation may provide valuable tools for the design of inhibitors of the assembly between PR and EZH2 which may hold therapeutic value.

The role of PcG in cell differentiation through repression of target genes is well acknowledged. The regulation of EZH2 by progesterone in the mammary gland [25] suggests that its activity may be linked to the action of hormone receptors. Therefore, EZH2 may be directed to a subset of target genes by hormonal cues. Regulation of the master transcription factor GATA3 through PR and EZH2 co-recruitment is in accordance with the reported role of PcG in cell fate transitions through regulation of genes involved in cell differentiation [24], as is the case of GATA3. It would be of interest to determine which sites are bound by both PR and EZH2 in normal mammary gland and in breast cancer cells by the use of genome-wide approaches, potentially allowing the identification of target genes repressed by PR in an EZH2-dependent manner. If the transcriptional repression described here for the *GATA3* locus proves to be a general mechanism for PR-mediated target gene repression, it could set the rationale for the use of EZH2 inhibitors in order to prevent PR-driven breast cancer progression, by blocking the repression of tumor suppressor genes and promoting a more differentiated status of breast cancer cells.

### Cyclin A2 regulation by GATA3

Cell cycle transitions are controlled by cyclin-dependent kinases (CDKs), whose activity depends on the interaction with specific cyclins which have distinctive expression patterns during progression of the cell cycle. Particularly, cyclin D1 expression peaks during the G1 phase of the cell cycle and interacts with Cdk2, Cdk4 and Cdk6 promoting the progression of the G1 phase of the cell cycle. Furthermore, cyclin A2 is expressed in proliferative somatic cells and its expression peaks during the S and G2 phases of the cell cycle, since it mediates the onset of DNA replication and, therefore, the transition from G1 to S phase of the cell cycle [28]. The results presented here suggest that GATA3 blockade of MPA-induced proliferation may involve direct cyclin A2 transcriptional repression. The close participation of GATA3 in mammary luminal cell fate favors the possibility of GATA3 regulating cell cycle progression. Indeed, several reports demonstrate GATA3 transcriptional regulation of factors which have pivotal functions in cell cycle control, for example, p18 (ink4c) [51],[52] and cyclin D1 [53],[54] where GATA3 binds to its responsive element in the respective promoters. Notably, the human cyclin A2 proximal promoter contains three GATA3 binding sites, two of them located in tandem at position -171 bp and another one mapped at -2,496 bp [42]. Further research is required to evaluate whether GATA3 alone, or as part of an enhanceosome, is recruited to these binding sites to repress MPA-induced cyclin A2 transcription.

On the one hand, GATA3 has been reported to be required for cyclin D1 expression [54] and on the other hand, we show here that GATA3 downregulation is needed for progestin-induced cyclin A2 levels. Notably, MPA phosphorylation of GATA3 at Ser 308 is induced at S/G2 suggesting that this event may precede cyclin A2 increase by progestin. These results indicate that GATA3 may coordinate the expression of these two cyclins. Our present demonstration of GATA3 regulation at both the transcriptional and post-translational levels may indicate the importance of the temporal coordination of its expression in order to synchronize the expression patterns of both cyclin D1 and cyclin A2. The two independent mechanisms described here for GATA3 downregulation may provide fine tuning to GATA3 expression. Further insight into the role of GATA3 in coordinating cell cycle progression may contribute to clarify its role in breast cancer.

### Relevance of GATA3 phosphorylation as a potential prognosis marker

Several studies demonstrated that GATA3 expression holds independent prognostic value of a favorable outcome in breast cancer patients [15]-[17],[19]. In the present work, we show the role of GATA3 phosphorylation at serine 308 for PR-induced GATA3 protein turnover. We have also shown that the capability of the non-phosphorylable mutant GFP-GATA3-S308A to prevent MPA-induced breast cancer cell proliferation is enhanced compared to its wild-type counterpart, as demonstrated in the *in vivo* assay in the C4HD preclinical model. Given the presented results establishing a link between GATA3 phosphorylation and increased protein turnover, we propose that determining pSer308-GATA3 levels in breast cancer patients could be predictive of GATA3 loss. The detection of pSer308-GATA3 could allow stratification of GATA3 positive tumors and identification of those predicted to lose GATA3 expression, which would be expected to bear a worse prognosis. The latter group of patients could benefit from antiprogestin treatment to prevent GATA3 degradation and hence prevent progestin-induced tumor growth.

## Conclusions

The results of this study demonstrate that progestins downregulate GATA3 transcript through PR binding upstream of the *GATA3* gene, EZH2 recruitment and chromatin compaction. We have also shown that PR activation leads to PKA-mediated GATA3 phosphorylation at serine 308, which results in 26S proteasome-mediated GATA3 protein degradation. Finally, we demonstrated that GATA3 downregulation is essential for cyclin A2 upregulation and progestin-driven breast cancer growth.

## Additional file

## Electronic supplementary material

Additional file 1: Supplemental data. Contains all supplemental figures and tables cited in this article. (PDF 6 MB)

Below are the links to the authors’ original submitted files for images.

Authors’ original file for figure 1

Authors’ original file for figure 2

Authors’ original file for figure 3

Authors’ original file for figure 4

Authors’ original file for figure 5

Authors’ original file for figure 6

## Abbreviations

ActD: actinomycin D
ANOVA: analysis of variance
bp: base pair
Btz: bortezomib
chFCS: charcoal-stripped FCS
ChIP: chromatin immunoprecipitation
CHX: cycloheximide
(D)MEM: (Dulbecco’s) modified Eagle’s medium
ER: estrogen receptor
EZH2: enhancer of zeste homolog 2
FCS: fetal calf serum
GFP: green fluorescent protein
IHC: immunohistochemistry
MFI: mean fluorescence intensity
MMTV: mouse mammary tumor virus
MPA: medroxyprogesterone acetate
PB: permeabilization buffer
PBS: phosphate-buffered saline
PcG: polycomb group G
PKA: protein kinase
PKI: protein kinase inhibitor
PR: progesterone receptor
PRE: progesterone response element
RT-qPCR: reverse transcriptase quantitative polymerase chain reaction
siRNA: small interfering RNA
TSS: translational start site
WT: wild type
