# Supplementary material for: Progesterone receptor activation downregulates GATA3 by transcriptional repression and increased protein turnover promoting breast tumor growth
Source: Breast Cancer Res. 2014 Dec 6;16:491. doi: 10.1186/s13058-014-0491-x (PMC4303201; doi:10.1186/s13058-014-0491-x)
Supplement: Supplementary file 1 — Additional file 1: Supplemental data. Contains all supplemental figures and tables cited in this article. (PDF 6 MB) [file 13058_2014_491_MOESM1_ESM.pdf]

SUPPLEMENTAL DATA

Progesterone Receptor Activation Downregulates GATA3 by Transcriptional Repression and Increased Protein Turnover Promoting Breast Tumor Growth

Franco Izzo, Florencia Mercogliano, Leandro Venturutti, Mercedes Tkach, Gloria Inurrigarro, Roxana Schillaci, Leandro Cerchietti, Patricia V Elizalde and Cecilia J Proietti.

Inventory of Supplemental Data

Figure S1,
Figure S2,
Figure S3,
Figure S4,
Figure S5,
Figure S6,
Figure S7,
Figure S8,
Figure S9,
Figure S10,
Figure S11,
Figure S12,
Figure S13.

Table S1,
Table S2.

Figure S1

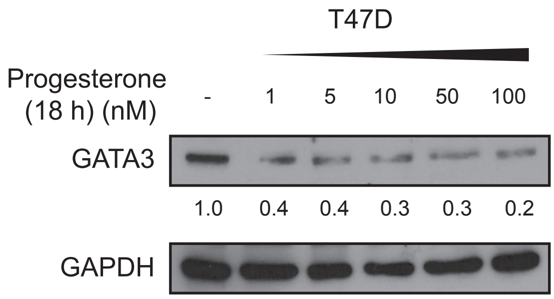

Figure S1. T47D cells were treated or not with the indicated concentrations of progesterone for 18 h. Total protein lysates were prepared and GATA3 expression was measured by western blot (WB). GATA3 bands underwent densitometry and values were normalized to GAPDH protein bands setting the value of untreated cells as 1.0.

Figure S2

ATTCAGCCCTGATTTCAAACAAACATCAGATTTAAGAAGGTGAGGACTCCAAAGAAGATCCAAACGCGAG

ACCAGGAAGATTCACGTTTCCACTACTTCTTCCAATACATTTCACTAATCTTTCTACAGGGATAGTGTTT

TAAAACACGACGACCCTCACACACCAAGGTGAAGTTGAATATTGTTGTTGTTCCTTCACCGCATGAAAGG

PRE primer forward

AATCCTTCAGAACTTACTTTCAGGGACGGCT [**TCTTCT**] ATCTCGAATTTTTGCATTTGTAGGGAATTTT

PRE

ATGGGGTAGAGGAGCGAAGAGGGAACAATGTCTCCAATTCAAATAAAACCCCAAATTAAGCTCTCAGCCC

PRE primer reverse

CATCGGTGGGATAGCCTGCGGGGGAACCCACTCCCTCTCCTGGCAGCATTGCCCTGGGGGCGAGCAGAGA

GTGGATTTGGAGTCTTCTTTTCCCGCCTGACCCCTGCTCGCCCGAGCAGCATTTTCGGGCCAGCGGCTGC

AGCCACCCGGGCGCCGCTAGGGGATGCAGTGGCTCAAAGGACCGAGCGGGCGGTGCAGGTTGGAACCCGC

GGGGCGGACCAATCGCGGCTCGGCCACAGCCTCGCCCGCTGATTGGTCCCTCCAGGCCCCGCCCCCGCTC

GCCCCGCCCCTCTCGCTGGGGCGCCTCGGAGCCGCGTGCCCTCCGCCCCGGGGTGCCCATTGCGCAGAGC

GTGGCCTGGAGACCCGCGAGCCGGGGAAGGTCGCCGTGGAGTCCCGACCAGAGGCCGGGGTTGGGGTCGG

TGCAGACCGAGGGCTGGTTTCCTTGACTGTGGGAGAAACGCCGGGAGCCGGAGTAAGTAGGGCTCCGGGC

GGGGCGAAAGGAAAAGTTGGGTCCCTAGAGTGAAGACCGAGTTCTTTCTGTCCGTCTACACTGAGCGTAC

TCGGGGAATGAGTTAGAGCCAGTCTCTTCCTCCCCTCCCCCCTTCTCATCCCTCACTGTTGCCACTCAAG

TCAAAAGCACACATTGATTACAAATATTAGGTCTGGAAAGGGCAGCTGCAACAGCTGAAGCGTGTTCACT

CTGGGGGCTTGAGAGCGCAGAAGGCTCGGGAAAGAGGTGACAATGACAACAAAATTGACGCGGACGCTCC

AGTCAAAGGCATCTCCCCTTTATCCGATGACTCACCCTCTTAGGAAGTCGGCCCGAGAGGCAAATCTCAA

AATACCTTGACATGAAACATTTTGTTTTTCTGATCAATTTAACGCGCACGTTTCCCCACATCGATGCGCT

CTCCCAAACACCCTGCATTAGATCCTAATAATGATCCATGCGTGCCTATTTTTTAAAAGTCTGAAAAAGA

AAATTCTGCCCATCGAAATGAACTTCATGAATGGGGCAGGCTGGCTGCACCGGGACGGAATCGTCCACCC

GACCCGAATGAATTGGCAGGAGCCGCGGCCACATTTAAAGGGCCAGAGCGCGCGTTCCCTCCCGTCCGCC

CCCAAGCCCCGCGGGCCTCGCCCACCCTGCCCGCCGCCCCTCCGCCGGCGGCCGCCCTCTGCGGCGCCCC

TTTCCGGTCAGTGGAGGGGCGGGAGGAGGGGCGGGGGTGCGCGGGGCGGGGGGAGAAGTCCTGGAGCGGG

TTTGGGTTGCAGTTTCCTTGTGCCGGGGATCCTGTCCCCTACTCGCCAGCGCCAGGCTCCTCCCCCCCGG

CGCGGATGACACTAGAACCTCCTTAAGTTGCGTCGCGCCACAGCTGTCTGCGAACACTGAGCTGCCT**GGC**

GCCGTCTTGATACTTTCAGAAAGAATGCATTCCCTGT

TSS
GATA3

PRE: -1498 bp to -1504 bp from TSS

Figure S2. The genomic sequence upstream of the GATA3 transcriptional start site is shown. The putative PRE detected by *in silico* analysis is highlighted in red, and the sequences that match the primers used (PRE primer set) are shown. The position of the putative PRE relative to the TSS is detailed below.

Figure S3

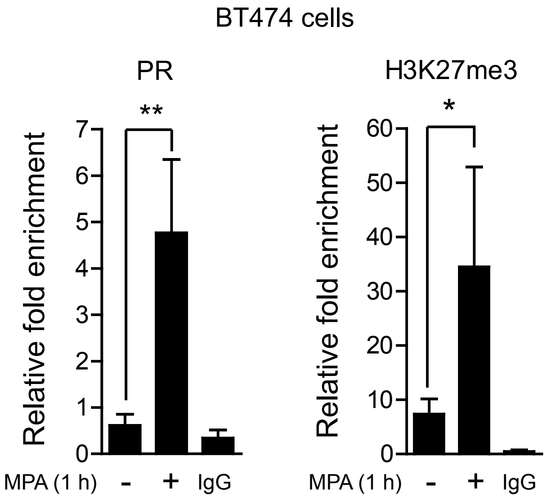

Figure S3. MPA induces PR recruitment to GATA3 promoter in BT474 cells. Protein recruitment to the GATA3 promoter was analyzed by ChIP in cells treated with MPA as indicated. Immunoprecipitated DNA was amplified by q-PCR using primers flanking the potential PRE located at position -1504 bp. Each sample was normalized to the input. Data are expressed as n-fold chromatin enrichment over isotype control (*P<0.05, **P<0.01, One-way ANOVA).

Figure S4

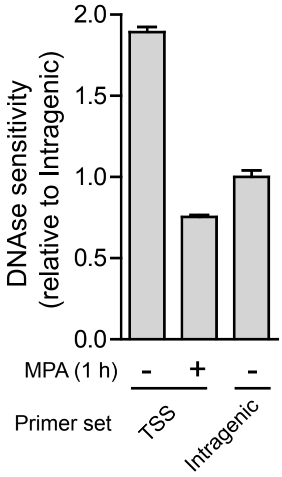

Figure S4. DNAse sensitivity assay was performed as described in the materials and methods section using primers spanning the region of *GATA3* transcriptional start site (TSS) or an intragenic region as control. Sensitivity was calculated as $2^{\wedge(Ctcut - Ctuncut)}$ for each treatment. One representative experiment of two is shown.

Figure S5

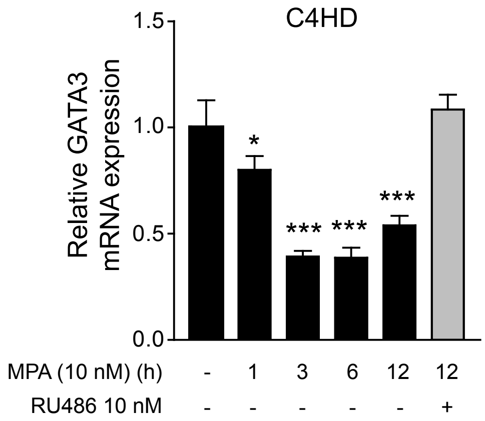

Figure S5. C4HD cells were treated as indicated and GATA3 mRNA expression levels were determined by RT-qPCR. The fold change of mRNA expression levels upon MPA treatment for the indicated times was calculated by normalizing the absolute levels of GATA3 mRNA to GAPDH levels, which were used as an internal control, and setting the value of untreated cells as 1.0. **P<0.01 and ***P<0.001 (One-way ANOVA).

Figure S6

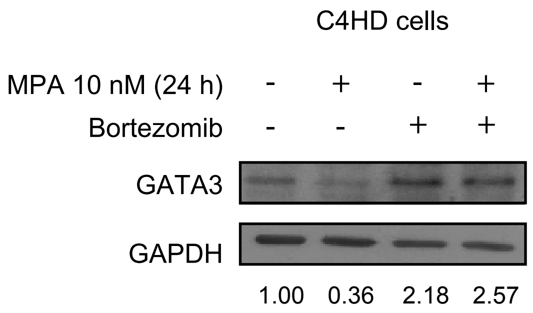

Figure S6. C4HD cells were treated or not with MPA and/or Bortezomib as indicated, protein cell lysates were prepared and GATA3 expression was measured by WB. GATA3 bands underwent densitometry and values were normalized to GAPDH protein bands setting the value of untreated cells as 1.0.

Figure S7

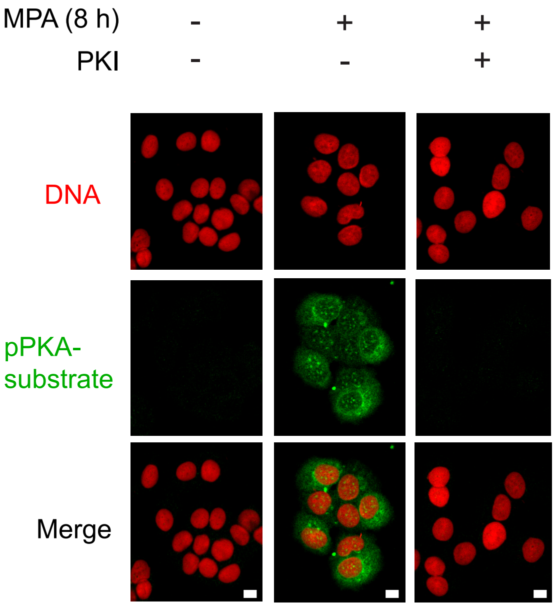

Figure S7. T47D cells were treated or not with MPA and/or the PKA inhibitor PKI for 8 h. Cells were then fixed and incubated with the indicated antibodies. Nuclei were stained with propidium iodide and confocal microscopy analysis was performed. Scale bar = 10 µm.

Figure S8

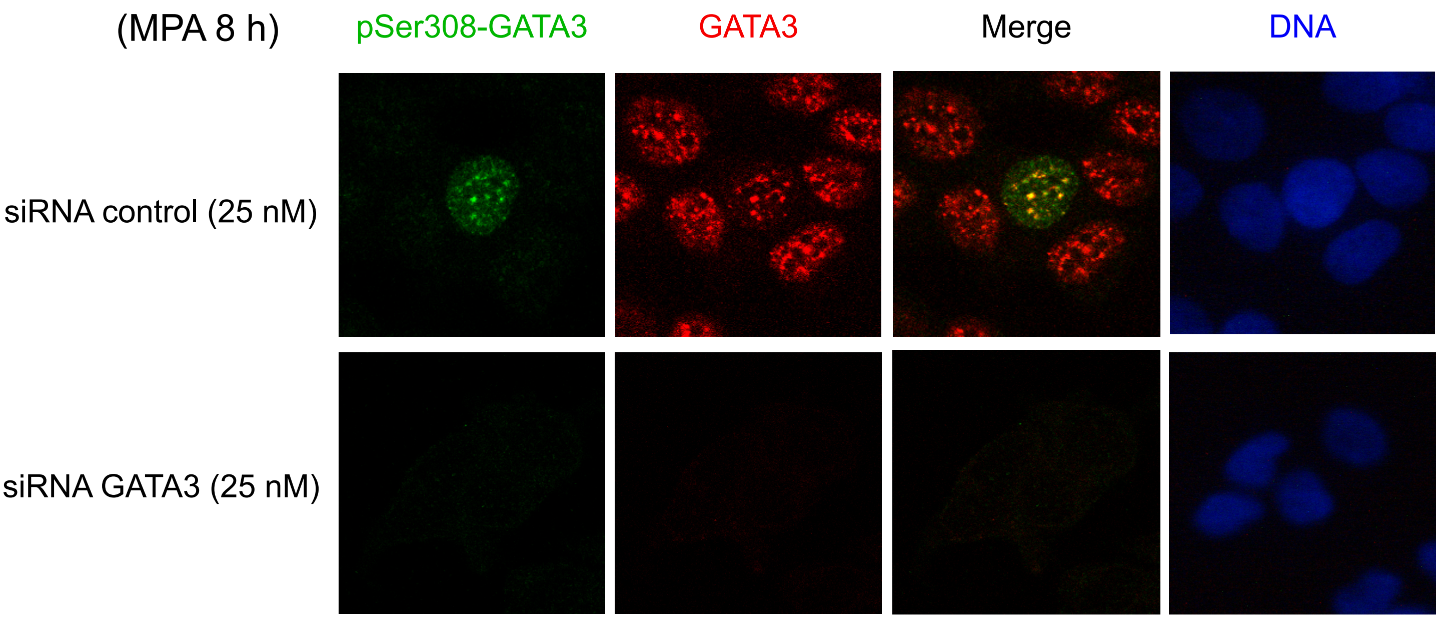

Figure S8. T47D cells were transfected with an unspecific siRNA as control or with a siRNA targeting GATA3 and treated with MPA for 8 h. Cells were fixed and pSer308-GATA3 and total GATA3 were analyzed by confocal microscopy. Nuclei were detected by DAPI staining.

Figure S9

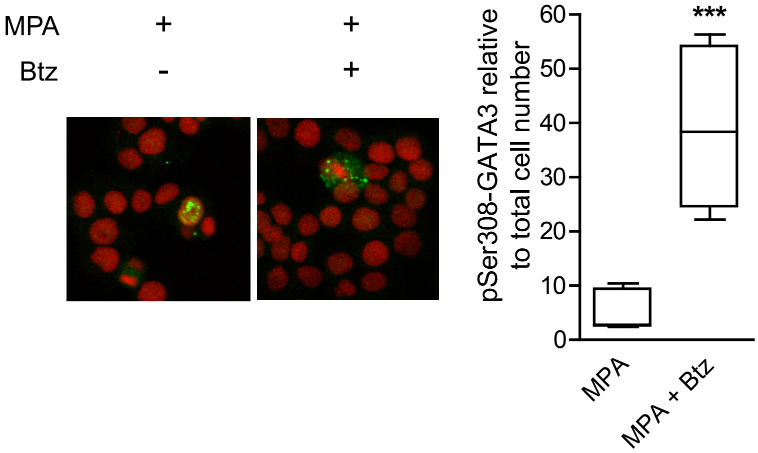

Figure S9. T47D cells were treated or not with MPA and Btz or 8 h. Cells were fixed and pSer308-GATA3 was analyzed by confocal microscopy. Nuclei were stained with propidium iodide. Scale bar = 10 µm. ***P<0.001 (Student's *t*-test).

Figure S10

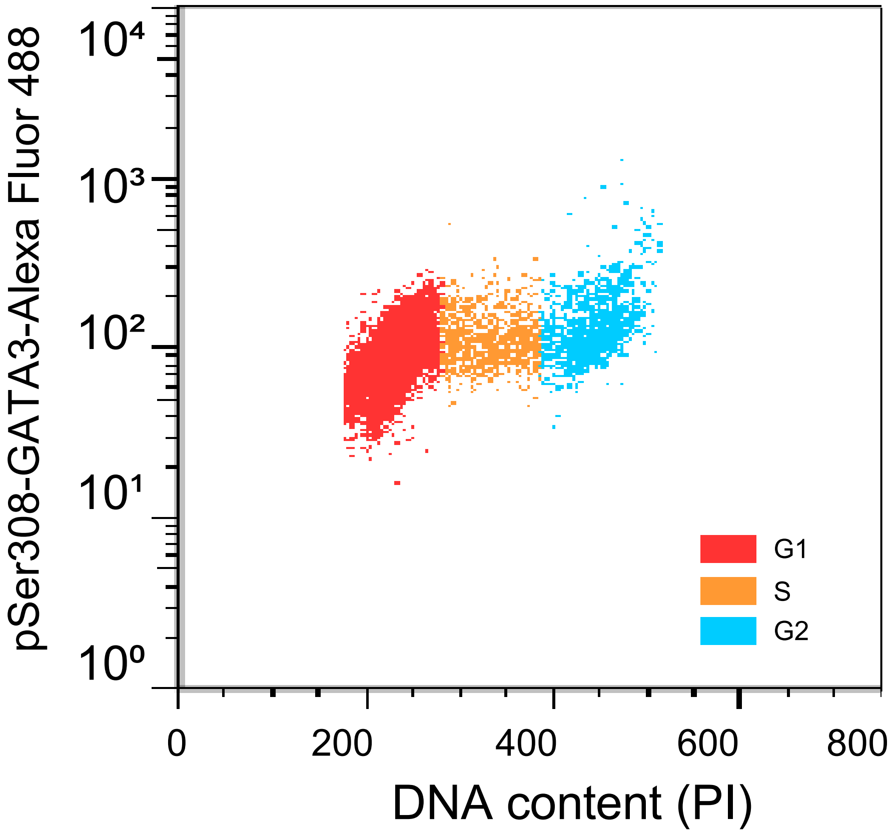

Figure S10.T47D cells treated with MPA for 8 h were stained with pSer308-GATA3-Alexa Fluor 488 and propidium iodide (PI) and were analyzed by flow cytometry. The different stages of the cell cycle were determined by PI. The experiment shown is representative of a total of three.

Figure S11

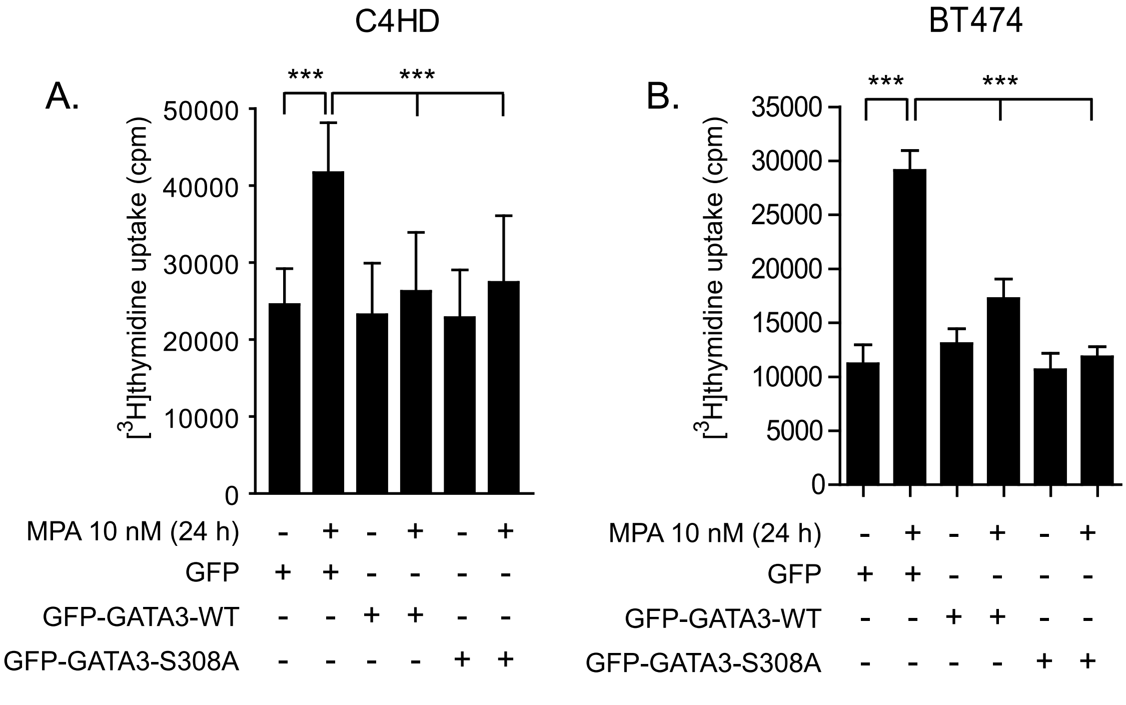

Figure S11. C4HD and BT474 cells were transfected with the indicated vectors for 48 h, starved for 24 h and treated with MPA as indicated. Incorporation of [^{3}H]-thymidine was used as measure of DNA synthesis and cell proliferation. ***P<0.001 (One-way ANOVA).

Figure S12

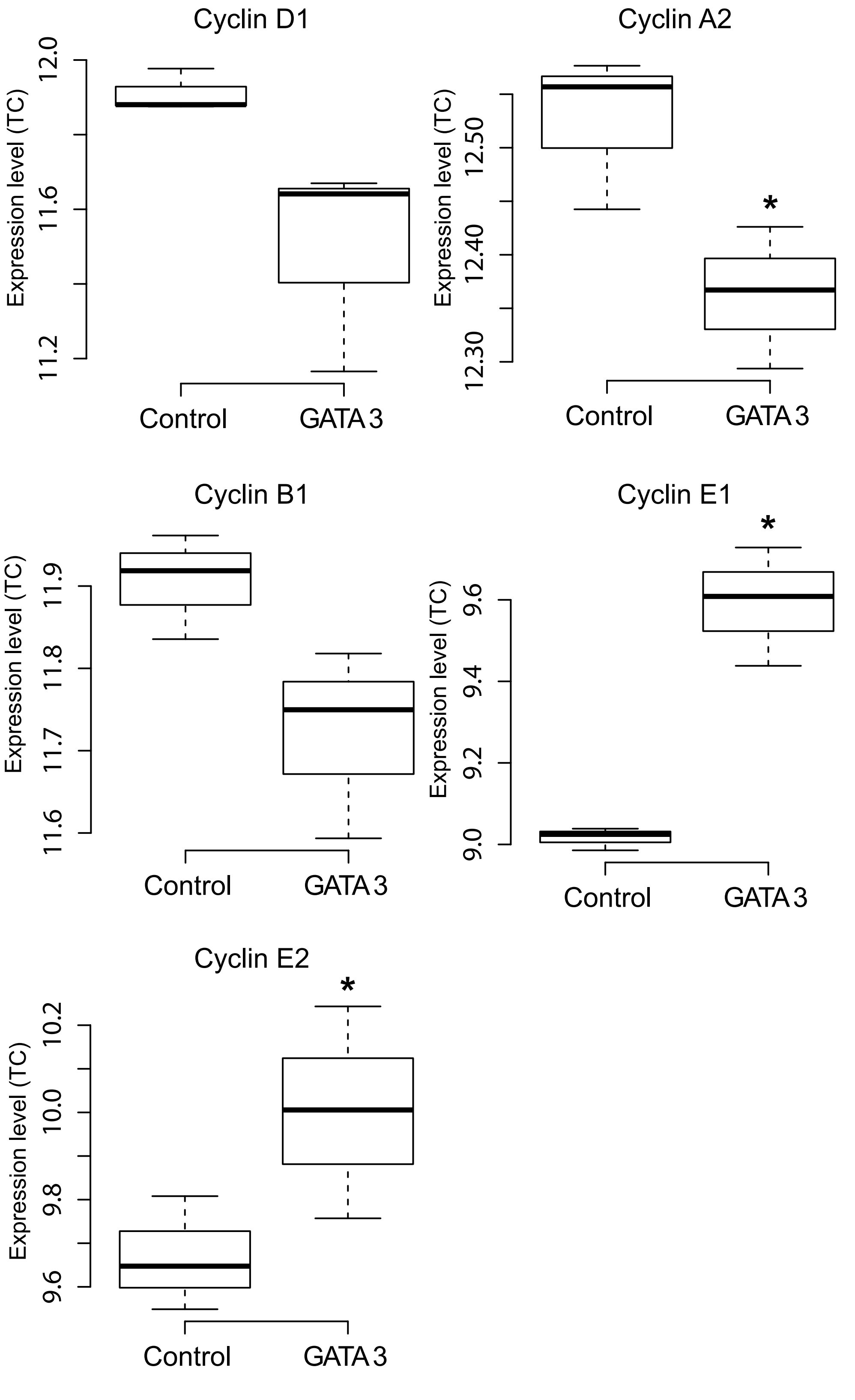

Figure S12. Data for the corresponding genes were downloaded from the publicly available Gene Expression Omnibus Data Sets (GDS4080) database and displayed as boxplots for the corresponding genes, comparing triplicate measures of transcriptional counts (TC). *P<0.05 (Student's *t*-test).

Figure S13

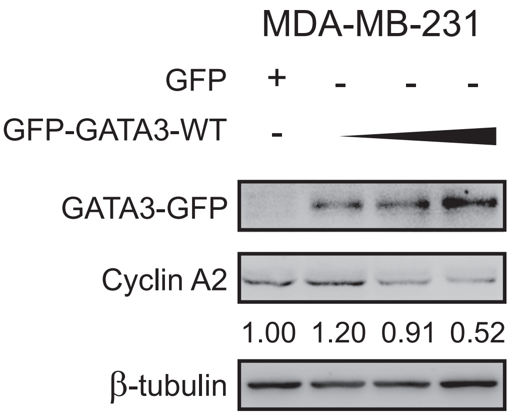

Figure S13. MDA-MB-231 cells were transfected with the indicated vectors for 48 h, protein lysates were prepared and immunoblotted for the indicated proteins. The densitometry values were obtained by normalizing cyclin A to GAPDH protein bands and setting the value of untreated cells as 1.0.

Supplementary Table 1

Primer	Sequence (5´- 3´)
PRE Forward	GACGACCCTCACACACCAAG
PRE Reverse	TCTTCGCTCCTCTACCCCAT
GATA3 Intragenic Forward	GGGAGAGCTCGTGGTATCTG
GATA3 Intragenic Reverse	TTTCTCCCCTCCATGTTCAG
GATA3 TSS Forward	TTGGGTTGCAGTTTCCTTGT
GATA3 TSS Reverse	CGACGCAACTTAAGGTT
GATA3 mRNA (human) Forward	CAGACCACCACAACCACACTCT
GATA3 mRNA (human) Reverse	GGATGCCTTCCTTCTTCATAGTCA
GATA3 mRNA (mouse) Forward	CTACCGGGTTCGGATGTAAGTC
GATA3 mRNA (mouse) Reverse	GTTCACACACTCCCTGCCTTCT
GAPDH mRNA Forward	CCAGAACATCATCCCTGCAT
GAPDH mRNA Reverse	GTTCAGCTCTGGGATGACCTT
Mutagenesis S308A Forward	CAAGCGAAGGGCTGGCTGCAGCCAGGAG
Mutagenesis S308A Reverse	CTCCTGTGGCTGCAGCCAGCCTTCGCTTG

Supplementary Table 2

siRNA	Sequence (5´-3´)
PR #1 (human)	CUAACAAAUCAUCGACUUA
PR #2 (human)	GUAGUCAAGUGGUCUAAAU
PR #1 (mouse)	CTCCACCTGTACTGCTTGAAT
PR #2 (mouse)	CTCCACCTGTACTGCTTGAAT
GATA3 (human)	AUAUCCAUCGCGUUUAGGCUU
