# Supplementary figures and images for: Progesterone receptor activation downregulates GATA3 by transcriptional repression and increased protein turnover promoting breast tumor growth
Source: Breast Cancer Res. 2014 Dec 6;16:491. doi: 10.1186/s13058-014-0491-x (PMC4303201; doi:10.1186/s13058-014-0491-x)

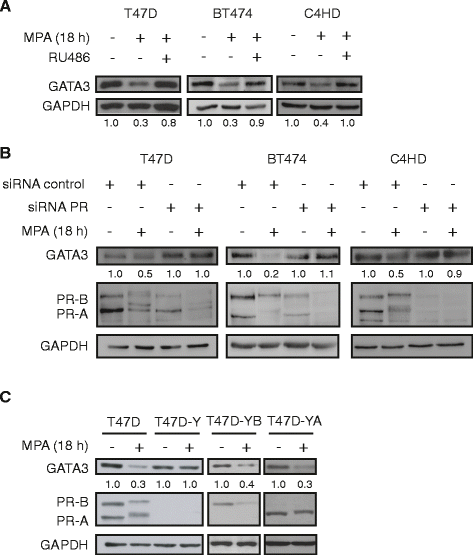

Supplement: Supplementary file 2 — Authors’ original file for figure 1 [file 13058_2014_491_MOESM2_ESM.gif]

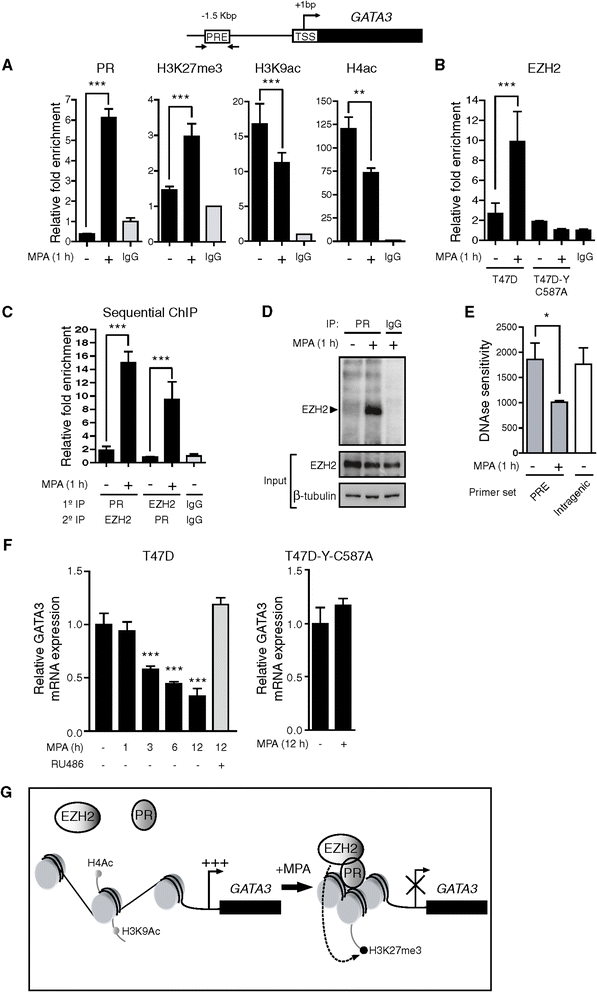

Supplement: Supplementary file 3 — Authors’ original file for figure 2 [file 13058_2014_491_MOESM3_ESM.gif]

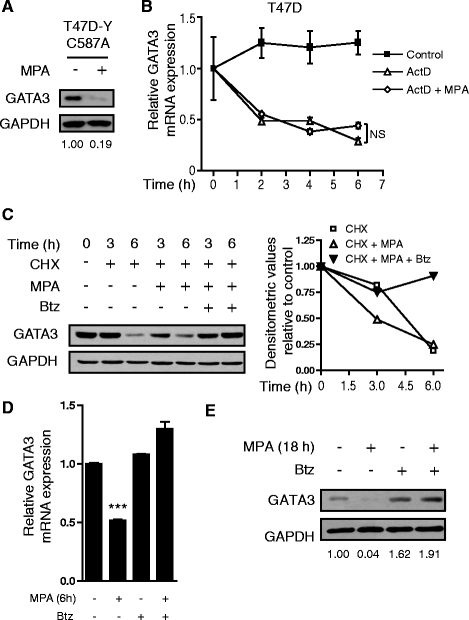

Supplement: Supplementary file 4 — Authors’ original file for figure 3 [file 13058_2014_491_MOESM4_ESM.gif]

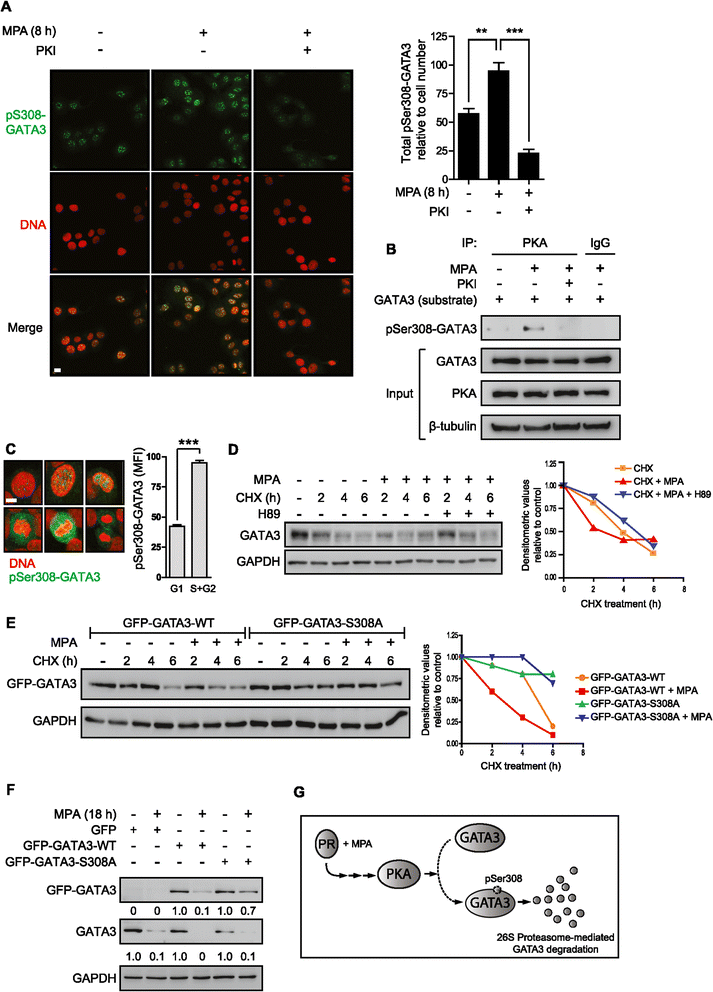

Supplement: Supplementary file 5 — Authors’ original file for figure 4 [file 13058_2014_491_MOESM5_ESM.gif]

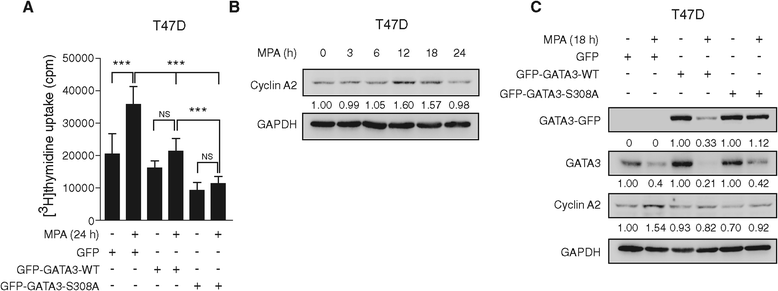

Supplement: Supplementary file 6 — Authors’ original file for figure 5 [file 13058_2014_491_MOESM6_ESM.gif]

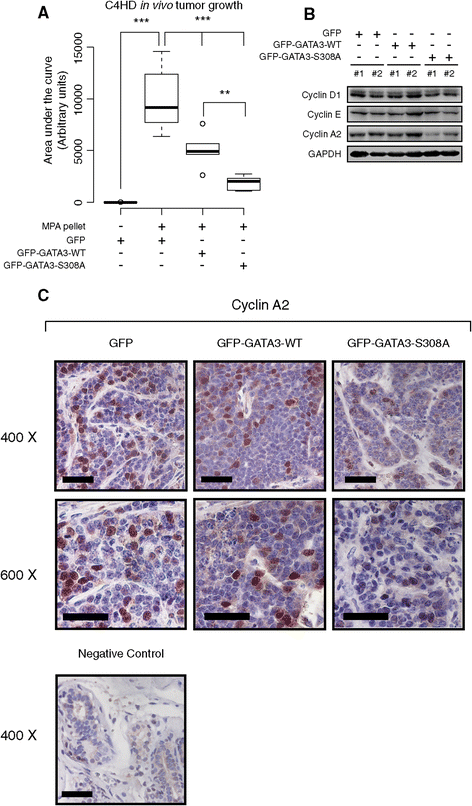

Supplement: Supplementary file 7 — Authors’ original file for figure 6 [file 13058_2014_491_MOESM7_ESM.gif]
